# Association of Achieving Time in Range Clinical Targets With Treatment Modality Among Youths With Type 1 Diabetes

**DOI:** 10.1001/jamanetworkopen.2023.0077

**Published:** 2023-02-20

**Authors:** Klemen Dovc, Stefanie Lanzinger, Roque Cardona-Hernandez, Martin Tauschmann, Marco Marigliano, Valentino Cherubini, Romualdas Preikša, Ulrike Schierloh, Helen Clapin, Fahed AlJaser, Julie Pelicand, Rishi Shukla, Torben Biester

**Affiliations:** 1 Department of Pediatric Endocrinology, Diabetes and Metabolic Diseases, University Children’s Hospital, Ljubljana, Slovenia; 2 Faculty of Medicine, University of Ljubljana, Ljubljana, Slovenia; 3 Institute of Epidemiology and Medical Biometry, ZIBMT, Ulm University, Ulm, Germany; 4 German Center for Diabetes Research (DZD), Munich–Neuherberg, Germany; 5 Division of Pediatric Endocrinology, Hospital Sant Joan de Déu, Barcelona, Spain; 6 Department of Pediatrics and Adolescent Medicine, Medical University of Vienna, Vienna, Austria; 7 Pediatric Diabetes and Metabolic Disorders Unit, Regional Center for Pediatric Diabetes, University City Hospital of Verona, Verona, Italy; 8 Department of Surgery, Dentistry, Paediatrics and Gynaecology, University of Verona, Verona, Italy; 9 Division of Pediatric Diabetology, Department of Women’s and Children’s Health, Salesi Hospital, Ancona, Italy; 10 Institute and Clinic of Endocrinology, Lithuanian University of Health Sciences, Kaunas; 11 Department of Pediatric Diabetes and Endocrinology, Centre Hospitalier Luxembourg, Luxembourg, Luxembourg; 12 Department of Diabetes and Endocrinology, Perth Children’s Hospital, Perth, Australia; 13 Department of Pediatrics, Amiri Hospital, Ministry of Health, Dasman, Kuwait; 14 Pediatric and Adolescent Diabetes Program, Department of Pediatrics, San Camilo Hospital, San Felipe, Chile; 15 Medicine School, Universidad de Valparaiso, San Felipe, Chile; 16 Department of Diabetes and Endocrinology, Center for Diabetes & Endocrine Diseases, Kanpur, India; 17 Children’s Hospital, Auf der Bult, Hannover, Germany

## Abstract

**Importance:**

Continuous glucose monitoring (CGM) devices have demonstrated efficacy in adults and more recently in youths and older adults with type 1 diabetes. In adults with type 1 diabetes, the use of real-time CGM compared with intermittently scanned CGM was associated with improved glycemic control, but there are limited data available for youths.

**Objective:**

To assess real-world data on achievement of time in range clinical targets associated with different treatment modalities in youths with type 1 diabetes.

**Design, Setting, and Participants:**

This multinational cohort study included children, adolescents, and young adults younger than 21 years (hereinafter referred to collectively as youths) with type 1 diabetes for a duration of at least 6 months who provided CGM data between January 1, 2016, and December 31, 2021. Participants were enrolled from the international Better Control in Pediatric and Adolescent Diabetes: Working to Create Centers of Reference (SWEET) registry. Data from 21 countries were included. Participants were divided into 4 treatment modalities: intermittently scanned CGM with or without insulin pump use and real-time CGM with or without insulin pump use.

**Exposures:**

Type 1 diabetes and the use of CGM with or without an insulin pump.

**Main Outcomes and Measures:**

Proportion of individuals in each treatment modality group achieving recommended CGM clinical targets.

**Results:**

Among the 5219 participants (2714 [52.0%] male; median age, 14.4 [IQR, 11.2-17.1] years), median duration of diabetes was 5.2 (IQR, 2.7-8.7) years and median hemoglobin A_1c_ level was 7.4% (IQR, 6.8%-8.0%). Treatment modality was associated with the proportion of individuals achieving recommended clinical targets. Adjusted for sex, age, diabetes duration, and body mass index standard deviation score, the proportion achieving the recommended greater than 70% time in range target was highest with real-time CGM plus insulin pump use (36.2% [95% CI, 33.9%-38.4%]), followed by real-time CGM plus injection use (20.9% [95% CI, 18.0%-24.1%]), intermittently scanned CGM plus injection use (12.5% [95% CI, 10.7%-14.4%]), and intermittently scanned CGM plus insulin pump use (11.3% [95% CI, 9.2%-13.8%]) (*P* < .001). Similar trends were observed for less than 25% time above (real-time CGM plus insulin pump, 32.5% [95% CI, 30.4%-34.7%]; intermittently scanned CGM plus insulin pump, 12.8% [95% CI, 10.6%-15.4%]; *P* < .001) and less than 4% time below range target (real-time CGM plus insulin pump, 73.1% [95% CI, 71.1%-75.0%]; intermittently scanned CGM plus insulin pump, 47.6% [95% CI, 44.1%-51.1%]; *P* < .001). Adjusted time in range was highest among real-time CGM plus insulin pump users (64.7% [95% CI, 62.6%-66.7%]). Treatment modality was associated with the proportion of participants experiencing severe hypoglycemia and diabetic ketoacidosis events.

**Conclusions and Relevance:**

In this multinational cohort study of youths with type 1 diabetes, concurrent use of real-time CGM and an insulin pump was associated with increased probability of achieving recommended clinical targets and time in range target as well as lower probability of severe adverse events compared with other treatment modalities.

## Key Points

**Question:**

Are different glucose monitoring and insulin delivery modalities associated with the achievement of recommended continuous glucose monitoring (CGM) targets in youths with type 1 diabetes?

**Findings:**

In this multinational cohort study including 5219 children, adolescents, and young adults with type 1 diabetes, the proportion of individuals achieving the recommended time in range target was associated with treatment modality. Users of real-time CGM concurrently with an insulin pump were the most likely to achieve the more than 70% time in, less than 25% time above, and less than 4% time below range clinical targets, had the adjusted highest time in range, and were the least likely to experience severe hypoglycemia and diabetic ketoacidosis events.

**Meaning:**

These findings suggest that for precise glycemic control and to achieve recommended clinical targets, real-time CGM used concurrently with an insulin pump should be more readily available to youths with type 1 diabetes.

## Teaser

This cohort study evaluates the the association of different treatment modalities with achieving recommended glucose levels in a large, diverse, international cohort of children, adolescents, and young adults with type 1 diabetes.

## Introduction

Precise life-long diabetes care is challenging for all individuals living with type 1 diabetes, yet imperative for the delay or prevention of long-term complications. There is increasing evidence that both glycemic excursions early in the disease course and high glucose concentrations have detrimental effects, suggesting it is critical to mitigate exposure to hyperglycemia and pronounced glucose level fluctuations.^1,2,3^ Regardless, achievement of glycemic targets remains elusive for most youths living with type 1 diabetes.^4^

The continued development of technology, including insulin pumps and continuous glucose monitoring (CGM) devices, has reformed the management of type 1 diabetes and offered the potential to optimize glycemic outcomes and improve quality of life.^5,6,7^ Two modalities of CGM are broadly available: real-time CGM, which continuously displays glucose concentration in the interstitial fluid (usually at intervals of 1-5 minutes) on a dedicated receiver or other portable device and provides various adjustable alarms, and intermittently scanned CGM, which displays data on demand when the transmitter is scanned using either a dedicated reader or smartphone-based application.

Since the inception of CGM, a large body of evidence from clinical trials has accumulated demonstrating efficacy of real-time and intermittently scanned CGM devices, especially if worn consistently.^8,9,10,11,12,13^ These results are further supported by findings from large international registries.^4,14,15,16,17^ Moreover, recent data support early adoption of CGM to improve glycemic outcomes and prevent acute complications soon after type 1 diabetes diagnosis.^14,18,19^

Less is known, however, about whether different CGM modalities, especially in conjunction with an insulin pump, provide any additional benefit in achieving recommended glycemic targets beyond monitoring hemoglobin A_1c_ (HbA_1c_) levels. Continuous glucose monitoring metrics, including time in range (70-180 mg/dL [to convert to mmol/L, multiply by 0.0555]), time below range (<70 mg/dL), and time above range (>180 mg/dL) targets^20^ can be used as relevant outcome measures or treatment targets, to provide guidance in daily decision-making and facilitate communication between people living with type 1 diabetes and their clinicians.^20,21,22^ The primary aim of this study was to evaluate the proportion of individuals achieving recommended CGM targets associated with different treatment modalities for diabetes care in a large, diverse, international cohort of children, adolescents, and young adults (hereafter referred to as youths) with type 1 diabetes from the international Better Control in Pediatric and Adolescent Diabetes: Working to Create Centers of Reference (SWEET) registry.

## Methods

SWEET is a worldwide network of centers providing care for children, adolescents, and young adults with diabetes. Its mission is to harmonize diabetes care, to optimize clinical outcomes, and thereby to establish standards of care in pediatric diabetes.^23^ The centers participating in the SWEET registry biannually export a set of standardized data, validated for credibility, to the Institute of Epidemiology and Medical Biometry, ZIBMT, Ulm University, Ulm, Germany. All contributing centers comply with current regulatory data protection security and ethics requirements. Verbal or written informed consent was obtained from individuals or their guardians based on national regulations in the respective countries. This study followed the Strengthening the Reporting of Observational Studies in Epidemiology (STROBE) reporting guideline.

### Trial Design and Participants

As of December 2021, the SWEET database contained information on 95 090 participants from 126 centers worldwide. In this multinational cohort study, data for SWEET participants younger than 21 years with type 1 diabetes for longer than 6 months who were using CGM and had at least 10 days of raw sensor data between January 1, 2016, and December 31, 2021, were included. Age, sex, diabetes duration, age at diabetes onset, total daily insulin dose, type of insulin administration (pump or injections), type of glucose monitoring (intermittently scanned or real-time CGM), HbA_1c_ level, and number of hospitalizations due to severe hypoglycemia or diabetic ketoacidosis were documented in the SWEET database. Race and ethnicity data are not included in the registry. Mean data from the earliest through the most recent date with CGM use were calculated and aggregated for each individual.^24^ Level of HbA_1c_ was standardized to the Diabetes Control and Complications Trial reference of 20 to 42 mmol/mol, or 4.0% to 6.0%.^25^ Severe hypoglycemia and diabetic ketoacidosis were defined according to International Society of Pediatric and Adolescent Diabetes Clinical Consensus Guidelines.^26,27^

### Outcomes

Outcomes were the proportion of individuals in each treatment modality group achieving recommended CGM-based targets (>70% time in range [70-180 mg/dL], <4% time below range [<70 mg/dL], and <25% time above range [>180 mg/dL]). Participants were categorized into 4 treatment groups depending on the glucose monitoring and insulin delivery modality: intermittently scanned CGM with insulin pump use, intermittently scanned CGM plus injection use, real-time CGM with insulin pump use, and real-time CGM plus injection use. Insulin pump included automated insulin delivery (AID) devices such as insulin suspension at or before low insulin levels and hybrid closed loop.

### Statistical Analysis

Continuous outcomes are presented as medians with IQRs and binary outcomes as numbers with percentages. For each of the 4 treatment modality categories, time in, above, and below range targets; HbA_1c_ level; and the proportion of participants with severe hypoglycemia and/or diabetic ketoacidosis during the observational period were calculated. We used Kruskal-Wallis and χ^2^ tests for unadjusted comparisons of the respective parameters. *P* values were adjusted for multiple comparisons with the false discovery rate method.

Sex, age (categorized), diabetes duration (categorized), body mass index standard deviation score (BMI-SDS; categorized) and treatment modality were included as covariates for all outcomes and accounted for center covariate as a random effect. Continuous covariables were categorized because of possible nonlinear associations between outcomes and covariables. Age was categorized as younger than 7 years, 7 to younger than 14 years, 14 to younger than 18 years, and 18 years or older. Diabetes duration was categorized as less than 2 years, 2 to less than 5 years, 5 to less than 10 years, and 10 years or longer. We derived the BMI-SDS from World Health Organization growth curves.^28^ The BMI-SDS was categorized as underweight (<–1.282), normal weight (–1.282 to 1.282), overweight (>1.282 to 1.881), and obese (>1.881).^24,28^ Fractional logistic regression models were used to investigate adjusted time in, above, and below the target range values. The proportion of individuals achieving CGM targets and the proportion of individuals with at least 1 severe hypoglycemia and/or diabetic ketoacidosis (treated as binary) event were studied using multivariable logistic regression models. We performed only complete case analyses, and participants with missing data were excluded from the regression models. Statistical analyses were conducted using SAS, version 9.4 (SAS Institute, Inc), and 2-sided *P* < .05 was considered statistically significant.

## Results

**Figure 1.**
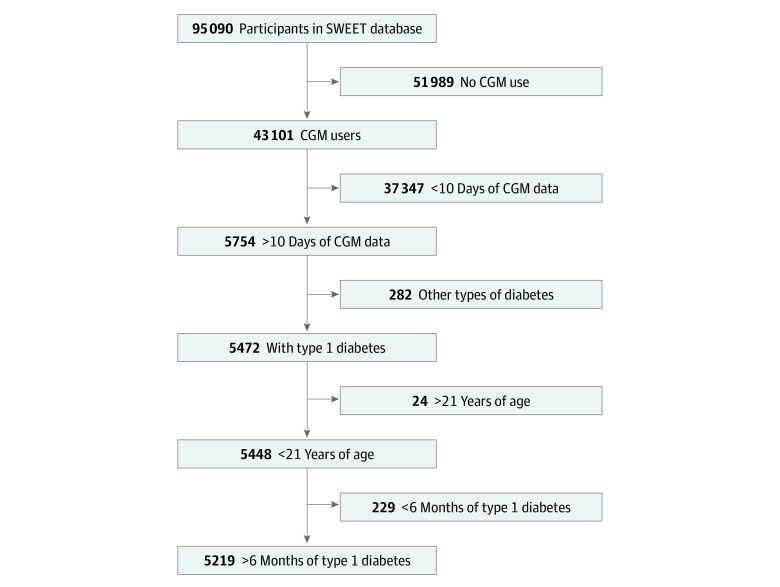
Study Flowchart CGM indicates continuous glucose monitoring; SWEET, Better Control in Pediatric and Adolescent Diabetes: Working to Create Centers of Reference.

A total of 5219 individuals from 34 participating centers and 21 countries were eligible for the study (Figure 1 and eTable 1 in Supplement 1). Demographic data, clinical characteristics, and unadjusted time in, above, and below range target, overall and stratified by treatment modality, are shown in the Table and the eFigure in Supplement 1. The median age was 14.4 (IQR, 11.2-17.1) years; median duration of diabetes, 5.2 (IQR, 2.7-8.7) years; and median HbA_1c_ level, 7.4% (IQR, 6.8%-8.0%) or 57 (IQR, 51-64) mmol/mol. A total of 2505 participants (48.0%) were female and 2714 (52.0%) were male. Participants had a median of 164 (IQR, 56-427) days of data available for this study. The overall median time in range was 59.3% (IQR, 47.0%-71.0%); time above range, 36.4% (IQR, 24.7%-48.9%); and time below range, 3.0% (IQR, 1.5%-5.3%). The distribution of treatment modalities was 850 participants (16.3%) using intermittently scanned CGM with and 1231 (23.6%) without an insulin pump, and 2252 (43.2%) using real-time CGM with and 886 (17.0%) without an insulin pump (Table).

**Table.** Baseline Characteristics of the Overall Study Population and Stratified by Treatment Modality^a^

Characteristic	Participant group				
	Overall (N = 5219)	Intermittently scanned CGM		Real-time CGM	
		With insulin pump use (n = 850)	With injection use (n = 1231)	With insulin pump use (n = 2252)	With injection use (n = 886)
Age, y	14.4 (11.2 to 17.1)	15.6 (12.8 to 17.7)	15.1 (12.3 to 17.4)	13.4 (10.3 to 16.1)	14.9 (11.4 to 17.6)
Age at type 1 diabetes onset, y	6.7 (3.8 to 10.3)	6.7 (3.9 to 10.0)	8.1 (5.7 to 11.7)	5.5 (2.8 to 8.7)	8.5 (5.1 to 11.6)
Duration of T1D, y	5.2 (2.7 to 8.7)	6.9 (4.1 to 10.2)	4.4 (2.4 to 7.3)	5.5 (2.9 to 8.8)	3.9 (1.7 to 7.7)
Sex, No. (%)					
Female	2505 (48.0)	450 (52.9)	584 (47.4)	1090 (48.4)	381 (43.0)
Male	2714 (52.0)	400 (47.1)	647 (52.6)	1162 (51.6)	505 (57.0)
BMI SDS^b^	0.6 (−0.1 to 1.2)	0.6 (0.1 to 1.3)	0.6 (−0.1 to 1.3)	0.5 (−0.1 to 1.2)	0.5 (−0.2 to 1.2)
HbA_1c_ level, %	7.4 (6.8 to 8.0)	7.5 (7.0 to 8.2)	7.5 (7.0 to 8.2)	7.2 (6.7 to 7.8)	7.4 (6.7 to 8.2)
HbA_1c_ level, mmol/mol	57 (51 to 64)	58 (53 to 66)	59 (53 to 66)	55 (50 to 62)	58 (50 to 66)
Time in target range of 70-180 mg/dL, %	59.3 (47.0 to 71.0)	51.7 (41.8 to 62.5)	53.8 (42.1 to 64.8)	65.4 (54.5 to 74.1)	59.0 (44.7 to 73.7)
Time above target range, %					
180 mg/dL	36.4 (24.7 to 48.9)	42.4 (32.2 to 53.5)	40.8 (29.7 to 53.0)	31.7 (21.7 to 42.6)	37.2 (22.3 to 52.4)
250 mg/dL	10.7 (4.8 to 20.4)	17.0 (9.4 to 27.7)	14.9 (6.8 to 25.7)	7.4 (3.5 to 14.3)	10.8 (4.1 to 21.9)
Time below target range, %					
70 mg/dL	3.0 (1.5 to 5.3)	4.3 (2.5 to 6.9)	3.9 (2.1 to 6.6)	2.4 (1.3 to 4.2)	2.5 (1.1 to 4.6)
54 mg/dL	0.5 (0.2 to 1.3)	1.0 (0.4 to 2.0)	0.7 (0.2 to 1.9)	0.4 (0.1 to 0.8)	0.3 (0.1 to 0.9)
Severe hypoglycemia, No. (%)	195 (3.7)	57 (6.7)	63 (5.1)	61 (2.7)	14 (1.6)
Diabetic ketoacidosis, No. (%)	97 (1.9)	35 (4.1)	20 (1.6)	37 (1.6)	5 (0.6)

Abbreviations: BMI SDS, body mass index standard deviation score; CGM, continuous glucose monitoring; HbA_1c_, hemoglobin A_1c_; T1D, type 1 diabetes.

^a^ Unless otherwise indicated, data are expressed as median (IQR). Data for the Better Control in Pediatric and Adolescent Diabetes: Working to Create Centers of Reference (SWEET) participants younger than 21 years with T1D duration of at least 6 months and who were using CGM were included from Australia, Austria, Chile, Croatia, Czech Republic, France, Germany, Greece, Hungary, India, Ireland, Italy, Kuwait, Lithuania, Luxembourg, Poland, Romania, Serbia, Slovenia, Spain, and Turkey. To convert mg/dL to mmol/L, multiply by 0.0555.

^b^ Calculated based on World Health Organization growth curves.^28^

Overall, 264 participants were classified in the database as using AID. AID users had higher unadjusted time in range (66.3% [IQR, 57.6%-75.8%] vs 59.0% [IQR, 46.6%-70.5%]; *P* < .001) and lower time above range (30.1% [IQR, 20.6%-39.1%] vs 37.0% [IQR, 25.1%-49.3%]; *P* < .001) compared with non-AID users (n = 4935). There was no difference in time below range (2.9% [95% CI, 1.7%-4.5%] vs 3.0% [95% CI, 1.5%-5,4%]; *P* = .46).

In the whole study cohort, 286 participants (5.5%) (median age, 15.4 [IQR, 12.9-17.4] years; 140 [49.0%] female and 146 [51.0%] male; median duration of diabetes, 6.6 [IQR, 4.5-10.2] years; median HbA_1c_ level, 7.7% [IQR, 7.2%-8.5%]) experienced at least 1 serious acute complication. These included 195 participants with severe hypoglycemia, 97 with diabetic ketoacidosis, and 9 with both. Their unadjusted time in range was 51.3% (95% CI, 40.2%-61.6%) compared with 59.9% (95% CI, 47.6%-71.4%; *P* < .001) of those who did not experience an acute complication during the observation period. Similarly, time above range was 43.3% (95% CI, 33.9%-55.4%) compared with 35.9% (95% CI, 24.3%-48.4%; *P* < .001). Time below range was 4.0% (95% CI, 2.4%-6.5%) compared with 2.9% (95% CI, 1.5%-5.3%; *P* < .001).

### Adjusted Logistic Regression Results

**Figure 2.**
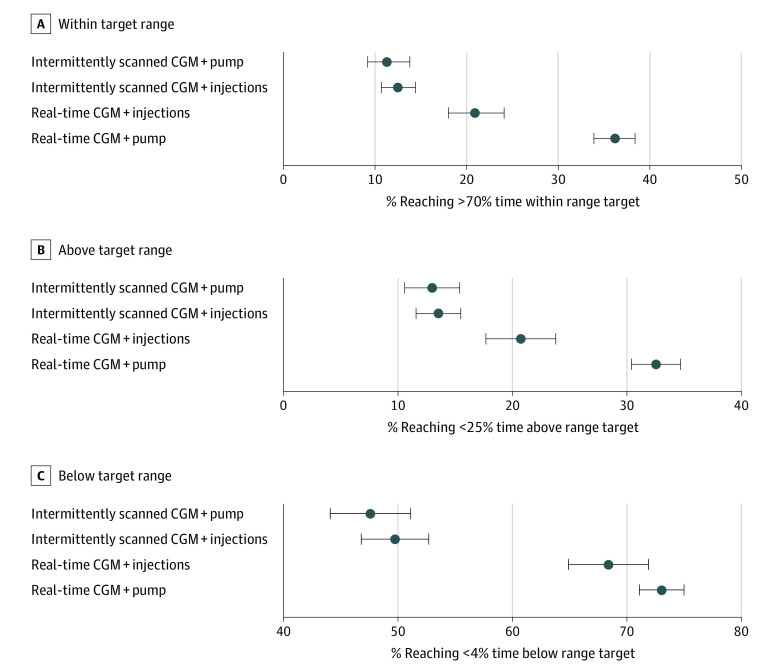
Proportions of Individuals With Type 1 Diabetes Reaching Recommended Targets in Association With Different Treatment Modalities Estimates with 95% CIs were derived from a logistic regression model adjusted for treatment modality, sex, age, diabetes duration, and body mass index standard deviation score for percentages of individuals reaching 70% time in the target range, 25% time above the target range, and 4% time below the target range. CGM indicates continuous glucose monitoring.

**Figure 3.**
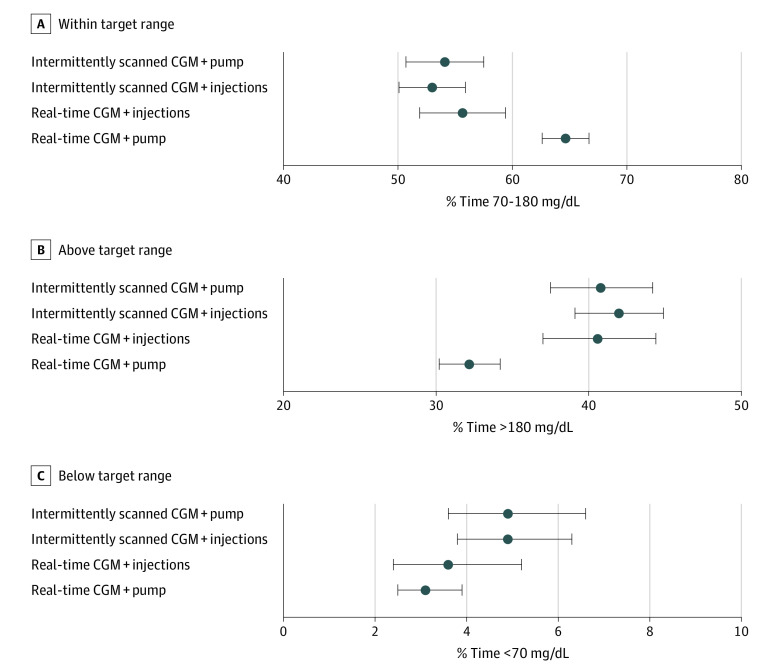
Proportions of Time in, Above, and Below the Target Range of Individuals With Type 1 Diabetes and Different Treatment Modalities Mean proportions with 95% CIs were derived from a fractional logistic regression model adjusted for treatment modality, sex, age, diabetes duration, and body mass index standard deviation score for time in the target range of glucose levels (70 to 180 mg/dL [to convert to mmol/L, multiply by 0.0555]), above the range (>180 mg/dL), and below the range (<70 mg/dL). CGM indicates continuous glucose monitoring.

The results of logistic regression and fractional logistic regression analyses are shown in Figure 2 and Figure 3 and eTables 2 and 3 in Supplement 1. When adjusted for sex, age, diabetes duration, and BMI-SDS, treatment modality as a covariate was associated with the probability of achieving recommended time in (70%), above (<25%), and below (<4%) range targets (Figures 2 and 3). Real-time CGM plus insulin pump use was the modality most likely to achieve recommended greater than 70% time in range clinical target (36.2% [95% CI, 33.9%-38.4%]), followed by real-time CGM plus injection use (20.9% [95% CI, 18.0%-24.1%]), intermittently scanned CGM plus injection use (12.5% [95% CI, 10.7%-14.4%]), and intermittently scanned CGM plus insulin pump use (11.3% [95% CI, 9.2%-13.8%]) (*P* < .001). Similar trends were observed for less than 25% time above range clinical target (32.5% [95% CI, 30.4%-34.7%] for real-time CGM plus insulin pump use, 20.6% [95% CI, 17.7%-23.8%] for real-time CGM plus injection use, 13.4% [95% CI, 11.6%-15.5%] for intermittently scanned CGM plus injection use, and 12.8% [95% CI, 10.6%-15.4%] for intermittently scanned CGM plus pump use; *P* < .001) and less than 4% time below range clinical target, where the highest proportions achieving recommended targets were observed (73.1% [95% CI, 71.1%-75.0%] for real-time CGM plus insulin pump use, 68.5% [95% CI, 64.9%-71.9%] for real-time CGM plus injection use, 49.7% [95% CI, 46.8%-52.7%] for intermittently scanned CGM plus injection use, and 47.6% [95% CI, 44.1%-51.1%] for intermittently scanned CGM plus pump use; *P* < .001).

Treatment modality was associated with time in range and time above range but not with time below range. The highest adjusted time in range was observed for real-time CGM plus insulin pump use (64.7% [95% CI, 62.6%-66.7%]), followed by real-time CGM plus injection use (55.7% [95% CI, 51.9%-59.4%]), intermittently scanned CGM plus insulin pump use (54.1% [95% CI, 50.7%-57.5%]), and intermittently scanned CGM plus injection use (53.0% [95% CI, 50.1%-55.9%]) (*P* < .001). Inversely, the lowest adjusted time above 250 mg/dL was observed for real-time CGM plus pump use (9.6% [95% CI, 8.4%-10.9%]), followed by real-time CGM plus injections use (16.2% [95% CI, 13.6%-19.2%]), intermittently scanned CGM plus pump use (17.6% [95% CI, 15.2%-20.3%]), and intermittently scanned CGM plus injections use (17.8% [95% CI, 15.7%-20.2%]) (*P* < .001). Similar results were observed for time above 180 mg/dL, while there was a smaller difference in time below range (Figures 2 and 3 and eTables 2 and 3 in Supplement 1).

Treatment modality as a covariate was associated with the probability of severe hypoglycemia and/or diabetic ketoacidosis events (eTable 4 in Supplement 1). The use of real-time CGM with or without an insulin pump was associated with a lower proportion of participants experiencing severe hypoglycemic events (2.5% [95% CI, 2.0%-3.3%] and 2.0% [95% CI, 1.2%-3.3%], respectively) compared with intermittently scanned CGM with and without insulin pump use (5.5% [95% CI, 4.1%-7.2%] and 5.2% [95% CI, 4.0%-6.6%], respectively) (*P* < .001). Similarly, the proportion of participants experiencing at least 1 diabetic ketoacidosis event varied from 1.4% (95% CI, 1.0%-2.0%) for real-time CGM plus insulin pump use and 0.7% (95% CI, 0.3%-1.6%) for real-time CGM plus injection use to 3.0% (95% CI, 2.0%-4.4%) for intermittently scanned CGM plus insulin pump use and 1.5% (95% CI, 1.0%-2.4%) for intermittently scanned CGM plus injection use (*P* = .001).

## Discussion

In this large real-world multinational cohort study of outcomes in youths with type 1 diabetes using different treatment modalities, concurrent real-time CGM and insulin pump use was associated with an approximately 15% higher proportion of individuals achieving the recommended greater than 70% time in range clinical target, and adjusted time in range was approximately 9% higher compared with youths using any other treatment combination. Furthermore, users of real-time CGM with an insulin pump were more likely to achieve the recommended times above and below range clinical targets,^21^ spent less time above range, and had a lower probability of experiencing severe adverse events. These results highlight the meaningful clinical impact of concurrent real-time CGM and insulin pump use in this population, which frequently struggles to achieve recommended glycemic targets.^11,29^ It is also encouraging that the unadjusted time in range achieved in the group with real-time CGM and insulin pump use was 65.4% (IQR, 54.5%-74.1%), approaching the consensus guideline targets of 70% for youths with type 1 diabetes. In a previous SWEET study,^29^ CGM use was associated with lower HbA_1c_ levels and fewer episodes of diabetic ketoacidosis requiring hospitalization compared with participants not using CGM.

Data from randomized clinical trials (RCTs) on the efficacy of intermittently scanned CGM compared with real-time CGM in individuals with type 1 diabetes are scarce and limited to the adult population. A study published in 2020 contrasted the use of real-time and intermittently scanned CGM among 60 adults with type 1 diabetes during 4 days of physical activity and an additional 4 weeks at home.^30^ There was a significant overall improvement in time in range (8.5%; *P* = .01), time above range (7.2%; *P* = .04), and time below range (2.9%; *P* = .006) with real-time CGM use. Recently, a large open-label, multicenter RCT contrasted real-time and intermittently scanned CGM use in a cohort of 254 adults with type 1 diabetes over 6 months (baseline HbA_1c_ level, 7.4% [58 mmol/mol]; 20% of whom were using an insulin pump) who previously used intermittently scanned CGM.^31^ During the observational period, time in range was improved by almost 7% (*P* < .001) with real-time CGM use, mainly due to reduced time above range (6.3%; *P* < .001). Importantly, real-time CGM users reported a better treatment satisfaction score at the end of the study.^31^ It is noteworthy that most included participants in both studies were treated with insulin injections.

While currently there are no data from RCTs comparing real-time and intermittently scanned CGM use in youths with type 1 diabetes, real-world data could provide some understanding regarding the association of glycemic outcomes with different treatment modalities in routine clinical care.^29,32,33^ In all evaluated treatment modalities, a much higher proportion of participants achieved time below range targets (45%-70%), compared to time in range and time above range clinical targets (10%-25%). There is accumulating evidence that youths with type 1 diabetes and parents of younger children with type 1 diabetes may use CGM to focus on avoiding hypoglycemia rather than improving time in range and preventing hyperglycemia.^34^ Nevertheless, the proportion of participants who achieved the consensus target of greater than 70% time in range increased by almost 15% with real-time CGM and insulin pump use (36.2%), compared with all other treatment modalities (20.9% with real-time CGM plus injection use, 12.5% with intermittently scanned CGM plus injection use, and 11.3% with intermittently scanned CGM plus insulin pump use). At the same time, approximately 12% more participants achieved targets less than 25% time above range and 5% more participants achieved targets less than 4% time below range with this treatment modality.

Duration of diabetes was the unmodifiable factor associated with both the achievement of recommended clinical targets and time in target values. As lifelong precise management is required, duration of diabetes is associated with disease fatigue, the burden of which could be eased by novel treatment modalities.^35^ In our analysis, the least favorable outcomes of achieving clinical targets were observed among the youngest participants (aged 1-7 years). One of the possible explanations could be that advanced glucose-responsive treatment modalities were less available in this age group than AID (only 1 AID system is currently approved in this age group), and reported use of AID systems was less than 1% in the youngest age group in our analysis.

In our study, additional glycemic benefits of insulin pump use were observed with real-time CGM use, but not intermittently scanned CGM use. Contrary to real-time CGM, the first generation of intermittently scanned CGM could not alert for high or low glucose values, which became available with the second and third generations of these devices. On the other hand, the real-time CGM automatically transmits a continuous stream of data to receiver and/or insulin pump and allows for the opportunity to semiautomate glucose-responsive insulin delivery, providing more physiological insulin replacement.^36^

Importantly, the use of real-time CGM was associated with a lower proportion of individuals with at least 1 severe hypoglycemic event when used with or without an insulin pump, compared with those using intermittently scanned CGM. Similar results were observed also for diabetic ketoacidosis episodes. Moreover, participants with at least 1 acute event (either severe hypoglycemia or diabetic ketoacidosis) had significantly lower time in range, further supporting the use of the time in range clinical target as a safe management tool in pediatric age groups.

### Strengths and Limitations

Strengths of this study include the heterogeneity of treatment modalities, the addition of glycemic metrics beyond HbA_1c_ levels, and diverse treatment data, data quality control, and broad worldwide representation, as participants were recruited from 34 centers and 21 countries within the SWEET registry. The challenges specific to the observational study design are the major limitations to this study. The study design limited our ability to collect socioeconomic and psychological data; therefore, we could not control for these possible confounders, as it is known that socioeconomic status is associated with access to the latest technical therapy.^37^ Furthermore, race and ethnicity are not documented in the SWEET database, as SWEET is an international registry including heterogeneous countries and regions. Therefore, it was not possible to give a standardized definition for race and ethnicity or racial and ethnic minority groups for the included countries. The study included centers from a variety of countries and systems providing health care that are not necessarily representative of the population, and centers with specialized expertise in the use of technologies were more likely to contribute raw sensor data. Multiple factors may have affected participants’ use of CGM modality and/or advanced pump technology, including availability, access, supplier assessment, and family preferences, and there is a clear need to address disparate outcomes for underserved communities.^38^ Currently, only a minority of CGM users in our registry provide CGM-level data. Lack of interoperability between manufacturers and/or third-party devices limits a direct integration of these data into electronic medical record systems and further into the SWEET database. Achieving interoperability, together with protecting data and device integrity privacy, could further improve the quality of our registry.^39^ While the SWEET registry includes data fields for newer generations of CGM devices, semiautomated functionalities that may be used by individuals combining real-time CGM with insulin pump use, these data were not automatically collected, and the numbers were likely underreported. The date of CGM initiation was not recorded.

## Conclusions

In this multinational cohort study of youths with type 1 diabetes, participants who were using real-time CGM with an insulin pump had a significantly higher time in range, were more likely to achieve recommended clinical targets, and had a lower proportion of severe adverse events compared with those using other treatment modalities. These results underscore the synergistic effect of advanced diabetes technologies that should be more readily available to youths with type 1 diabetes for further improvement of diabetes-related clinical outcomes.

## Notes

Supplement 1. **eMethods.** Study Consortia **eTable 1.** Baseline Characteristics of All CGM Users in the SWEET Database During the Observational Period **eTable 2.** Proportions of Individuals Reaching Recommended Targets in Association With Treatment Modality, Sex, Age, Diabetes Duration, and BMI-SDS **eTable 3.** Time in Range, Time Below Range, and Time Above Range in Association With Treatment Modality, Sex, Age, Diabetes Duration, and BMI-SDS **eTable 4.** Proportion of Individuals With ≥1 Severe Hypoglycemia and/or Diabetic Ketoacidosis Event in Association With Treatment Modality, Sex, Age, Diabetes Duration, and BMI-SDS **eFigure.** Unadjusted Median Glucose Concentrations Over the 24-Hour Period for Different Treatment Modalities

Supplement 2.
Data Sharing Statement
