# Supplementary material for: Association of Achieving Time in Range Clinical Targets With Treatment Modality Among Youths With Type 1 Diabetes
Source: JAMA Netw Open. 2023 Feb 20;6(2):e230077. doi: 10.1001/jamanetworkopen.2023.0077 (PMC9941889; doi:10.1001/jamanetworkopen.2023.0077)
Supplement: Supplement 1. — eMethods. Study Consortia eTable 1. Baseline Characteristics of All CGM Users in the SWEET Database During the Observational Period eTable 2. Proportions of Individuals Reaching Recommended Targets in Association With Treatment Modality, Sex, Age, Diabetes Duration, and BMI-SDS eTable 3. Time in Range, Time Below Range, and Time Above Range in Association With Treatment Modality, Sex, Age, Diabetes Duration, and BMI-SDS eTable 4. Proportion of Individuals With ≥1 Severe Hypoglycemia and/or Diabetic Ketoacidosis Event in Association With Treatment Modality, Sex, Age, Diabetes Duration, and BMI-SDS eFigure. Unadjusted Median Glucose Concentrations Over the 24-Hour Period for Different Treatment Modalities [file jamanetwopen-e230077-s001.pdf]

Supplementary Online Content

Dovc K, Lanzinger S, Cardona-Hernandez R, et al. Association of achieving time in range clinical targets with treatment modality among youths with type 1 diabetes. *JAMA Netw Open*. 2023;6(2):e230077. doi:10.1001/jamanetworkopen.2023.0077

eMethods. Study Consortia
eTable 1. Baseline Characteristics of All CGM Users in the SWEET Database During the Observational Period
eTable 2. Proportions of Individuals Reaching Recommended Clinical Targets in Association With Treatment Modality, Sex, Age, Diabetes Duration, and BMI-SDS
eTable 3. Time in Range, Time Below Range, and Time Above Range in Association With Treatment Modality, Sex, Age, Diabetes Duration, and BMI-SDS
eTable 4. Proportion of Individuals With ≥1 Severe Hypoglycemia and/or Diabetic Ketoacidosis Event in Association With Treatment Modality, Sex, Age, Diabetes Duration, and BMI-SDS
eFigure. Unadjusted Median Glucose Concentrations Over the 24-Hour Period for Different Treatment Modalities

This supplementary material has been provided by the authors to give readers additional information about their work.

© 2023 Dovc K et al. *JAMA Network Open*.

eMethods. Study Consortia

Australia, Perth: Princess Margaret Hospital for Children
Austria, Vienna: Universitätskinderklinik Wien
Chile, San Felipe: Hospital San Camilo
Croatia, Zagreb: University Clinical Hospital Center Sestre milosrdnice
Czech Republic, Prague: University Hospital Motol Prague
France, Angers: University Hospital Angers
Germany, Hannover: Kinderkrankenhaus Auf der Bult
Germany, Leverkusen: Klinikum Leverkusen - Kinderklinik
Greece, Athens: Athens University
Greece, Athens: P&A Kyriakou Childrens Hospital
Greece, Thessaloniki: AHEPA University Hospital, 2nd Department of Paediatrics, Aristotle University of Thessaloniki
Greece, Thessaloniki: Hippokration Hospital of Thessaloniki
Hungary, Budapest: Semmelweis University
India, Ahmedabad Bareja: Rudraksha Insitute of Medical Sciences
India, Ahmedabad: Arogyam Health Care
India, Kanpur: Center for Diabetes & Endocrine Diseases
Ireland, Cork: Cork University Hospital
Italy, Ancona: Salesi University Hospital
Italy, Florence: Meyer Children Hospital
Italy, Verona: Universisita di Verona
Kuwait, Kuwait City: Dasman Institute
Lithuania, Kaunas: Hospital of LUHS Kauno Klinikos
Luxembourg, Centre Hospitalier de Luxembourg
Poland, Katowicze: Medical University of Silesia
Poland, Lodz: Medical University
Poland, Opole: USK Opole
Poland, Rzeszow: University of Rzeszow, Pediatric Endocrinology and Diabetes
Poland, Warsaw: Medical University of Warsaw
Romania, Bucharest: Diabetes Nutrition and Metabolic Diseases Clinic DiabNutriMed
Serbia, Belgrade: Institute for Mother and Child Healthcare
Slovenia, Ljubljana: University Childrens Hospital
Spain, Barcelona: Hospital Sant Joan de Deu
Turkey, Duzce: University of Duzce,Department of Pediatric Endocrinology
Turkey, Ege University Faculty of Medicine

© 2023 Dovc K et al. *JAMA Network Open*.

eTable 1. Baseline Characteristics of All CGM Users in the SWEET Database During the Observational Period

Characteristics (n = 43,101)	Median (IQR)
Age (y)	14.7 (11.2, 17.6)
Age at type 1 diabetes onset (y)	7.9 (4.5, 11.2)
Duration of T1D (y)	5.3 (2.7, 8.8)
Female gender (%)	49.0
Body Mass Index SDS	0.6 (-0.1, 1.4)
HbA1c (%)	7.8 (6.9, 8.9)
HbA1c (mmol/mol)	61 (52, 74)

Data are median (IQR) or count (percentage).

BMI SDS – Body Mass Index Standard Deviation Score, calculated based on World Health Organization references, CGM – Continuous Glucose Monitoring,

© 2023 Dovc K et al. *JAMA Network Open*.

eTable 2. Proportions of Individuals Reaching Recommended Clinical Targets in Association With Treatment Modality, Sex, Age, Diabetes Duration, and BMI-SDS

**Variable**	**% of reaching Time in range (70–180 mg/dL) >70%***	**P***	**% of reaching Time below range 70mg/dL <4%***	**P***	**% of reaching Time above range 180 mg/dL <25%***	**P***
Treatment modality		<0.001		<0.001		<0.001
isCGM+pump	11.3 (9.2–13.8)		47.6 (44.1–51.1)		12.8 (10.6–15.4)	
isCGM+injections	12.5 (10.7–14.4)		49.7 (46.8–52.7)		13.4 (11.6–15.5)	
rtCGM+injections	20.9 (18.0–24.1)		68.5 (64.9–71.9)		20.6 (17.7–23.8)	
rtCGM+Pump	36.2 (33.9–38.4)		73.1 (71.1–75.0)		32.5 (30.4–34.7)	
Gender		0.115		0.031		0.049
Female	20.9 (19.2–22.8)		64.6 (62.6–66.6)		20.2 (18.5–22.0)	
Male	22.9 (21.1–24.7)		61.5 (59.5–63.5)		22.6 (20.9–24.4)	
Type 1 diabetes duration (y)		<0.001		<0.001		<0.001
0.5 – <2	55.8 (52.0–59.5)		75.1 (71.8–78.1)		51.4 (47.6–55.1)	
2 – <5	27.9 (25.4–30.3)		64.8 (62.2–67.2)		27.1 (24.8–29.5)	
5 – <10	13.8 (12.2–15.5)		57.0 (54.5–59.5)		13.6 (12.0–15.3)	
>10	10.6 (8.7–12.8)		58.9 (55.2–62.4)		11.5 (9.6–13.8)	
Age group (y)		<0.001		0.003		<0.001
1 – <7	11.8 (8.9–15.5)		60.5 (53.3–67.2)		11.8 (8.9–15.5)	
7 – <14	23.0 (21.0–25.1)		65.8 (63.5–68.0)		21.6 (19.3–23.7)	
14 – <18	22.2 (20.2–24.3)		62.6 (60.3–64.8)		21.4 (19.4–23.4)	
18 – <21	22.4 (9.1–26.1)		57.5 (53.7–61.3)		24.8 (21.4–28.6)	
BMI SDS		<0.001		0.004		<0.001
Underweight	24.0 (18.0–31.3)		65.9 (58.2–72.8)		20.2 (14.9–26.9)	
Normal	23.8 (22.2–25.4)		61.5 (59.9–63.2)		23.3 (21.8–24.8)	
Overweight	19.5 (16.6–22.9)		65.4 (61.6–69.1)		20.6 (17.6–24.0)	
Obese	12.9 (10.1–16.2)		69.5 (65.1–73.6)		11.7 (9.1–14.9)	

***Adjusted Estimates with 95% Confidence interval and P values were derived from logistic regression model, adjusted for treatment modality, gender, age, diabetes duration and BMI SDS.** BMI SDS – Body Mass Index Standard Deviation Score, calculated based on World Health Organization references, categorized as underweight (<– 1.282), normal weight (–1.282 – ≤ 1.282), overweight (>1.282 – ≤ 1.881) and obese (> 1.881).

CGM – Continuous Glucose Monitoring, isCGM – intermittently scanned CGM, rtCGM – real–time CGM

TAR – Time above range >180 mg/dl (10 mmol/l), TBR – Time below range <70 mg/dl (3.9 mmol/l), TIR – Time in range 70–180 mg/dl (3.9–10 mmol/l)

© 2023 Dovc K et al. *JAMA Network Open*.

eTable 3. Time in Range, Time Below Range, and Time Above Range in Association With Treatment Modality, Sex, Age, Diabetes Duration, and BMI-SDS

Variable	Time in range 70–180 mg/dL (%)*	P*	Time below range 70 mg/dL (%)*	P*	Time above range 180 mg/dL (%)*	P*
Treatment modality		<0.001		0.037		<0.001
isCGM with pump	54.1 (50.7–57.5)		4.9 (3.6–6.6)		40.8 (37.5–44.2)	
isCGM with injections	53.0 (50.1–55.9)		4.9 (3.8–6.3)		42.0 (39.1–44.9)	
rtCGM with injections	55.7 (51.9–59.4)		3.6 (2.4–5.3)		40.6 (37.0–44.4)	
rtCGM with pump	64.7 (62.6–66.7)		3.1 (2.5–3.9)		32.2 (30.2–34.2)	
Gender		0.563		0.672		0.450
Female	58.4 (56.4–60.4)		3.7 (3.0–4.6)		37.7 (35.7–39.7)	
Male	59.2 (57.3–61.2)		3.9 (3.3–4.8)		36.6 (34.8–38.6)	
Type 1 diabetes duration (y)		<.001		0.528		<0.001
0.5 – <2	70.2 (66.9–73.4)		3.0 (2.0–4.5)		26.7 (23.7–29.9)	
2 – <5	60.8 (58.3–63.3)		3.8 (2.9–4.9)		35.3 (32.9–37.8)	
5 – <10	54.8 (52.4–57.2)		4.2 (3.3–5.3)		40.8 (38.5–43.3)	
>10	51.7 (48.1–55.2)		4.2 (3.0–5.8)		44.2 (40.5–47.6)	
Age group (y)		0.486		0.820		0.541
1 – <7	54.4 (47.6–61.0)		4.2 (2.1–8.2)		41.2 (34.7–48.1)	
7 – <14	59.7 (57.4–61.9)		3.6 (2.8–4.5)		36.6 (34.4–38.8)	
14 – <18	58.4 (56.1–60.7)		3.8 (3.1–4.8)		37.6 (35.4–39.8)	
18 – <21	59.2 (55.5–62.8)		4.3 (3.1–6.1)		36.2 (32.7–39.9)	
BMI SDS		0.057		0.896		0.025
Underweight	60.3 (52.7–67.5)		3.4 (1.6–7.4)		36.1 (29.2–43.6)	
Normal	59.7 (58.0–61.3)		4.0 (3.4–4.7)		36.2 (34.6–37.8)	
Overweight	58.0 (54.2–61.8)		3.7 (2.5–5.4)		38.1 (34.5–41.9)	
Obese	53.1 (48.5–57.6)		3.3 (2.1–5.4)		43.4 (38.9–48.0)	

***Adjusted means with 95% Confidence interval and P values were derived from fractional logistic regression model, adjusted for treatment modality, gender, age, diabetes duration and BMI SDS.** BMI SDS – Body Mass Index Standard Deviation Score, calculated based on World Health Organization references, categorized as underweight (<– 1.282), normal weight (–1.282 – ≤ 1.282), overweight (>1.282 – ≤ 1.881) and obese (> 1.881).
CGM – Continuous Glucose Monitoring, isCGM – intermittently scanned CGM, rtCGM – real–time CGM

© 2023 Dovc K et al. *JAMA Network Open*.

eTable 4. Proportion of Individuals With ≥1 Severe Hypoglycemia and/or Diabetic Ketoacidosis Event in Association With Treatment Modality, Sex, Age, Diabetes Duration, and BMI-SDS

Variable	Hypoglycemic event (%)	P*	Diabetic ketoacidosis (%)	P*
Treatment modality		<0.001		0.001
isCGM with pump	5.5 (4.1–7.1)		2.9 (2.0–4.4)	
isCGM with injections	5.2 (4.0–6.6)		1.5 (1.0–2.4)	
rtCGM with injections	2.0 (1.2–3.4)		0.7 (0.3–1.6)	
rtCGM with pump	2.5 (1.9–3.3)		1.4 (1.0–2.0)	
Gender		0.980		0.590
Female	3.3 (2.6–4.1)		1.6 (1.1–2.1)	
Male	3.4 (2.7–4.2)		1.4 (1.0–1.9)	
Type 1 diabetes duration (y)		<0.001		<0.001
0.5 – <2	1.5 (0.8–2.6)		0.8 (0.4–1.7)	
2 – <5	2.8 (2.1–3.8)		0.9 (0.5–1.4)	
5 – <10	4.7 (3.6–5.6)		2.4 (1.8–3.3)	
>10	5.2(3.8–7.0)		2.5 (1.6–3.9)	
Age group (y)		0.205		0.013
1 – <7	4.4 (2.1–9.1)		2.8 (1.0–7.3)	
7 – <14	3.0 (2.3–3.9)		1.5 (1.0–2.1)	
14 – <18	3.5 (2.7–4.4)		2.0 (1.4–2.7)	
18 – <21	3.6 (2.5–5.1)		5.8 (0.2–1.2)	
BMI SDS		0.904		0.796
Underweight	3.0 (1.3–6.6)		1.4 (0.4–4.2)	
Normal	3.3 (2.7–3.9)		1.5 (1.1–2.0)	
Overweight	3.8 (2.6–5.5)		1.1 (0.5–2.1)	
Obese	3.5 (2.2–5.5)		1.6 (0.9–3.0)	

***Adjusted means with 95% Confidence interval and P values were derived from fractional logistic regression model, adjusted for treatment modality, gender, age, diabetes duration and BMI SDS.** BMI SDS – Body Mass Index Standard Deviation Score, calculated based on World Health Organization references, categorized as underweight (<– 1.282), normal weight (–1.282 – ≤ 1.282), overweight (>1.282 – ≤ 1.881) and obese (> 1.881).
CGM – Continuous Glucose Monitoring, isCGM – intermittently scanned CGM, rtCGM – real–time CGM

© 2023 Dovc K et al. *JAMA Network Open*.

eFigure 1. Unadjusted Median Glucose Concentrations Over the 24-Hour Period for Different Treatment Modalities

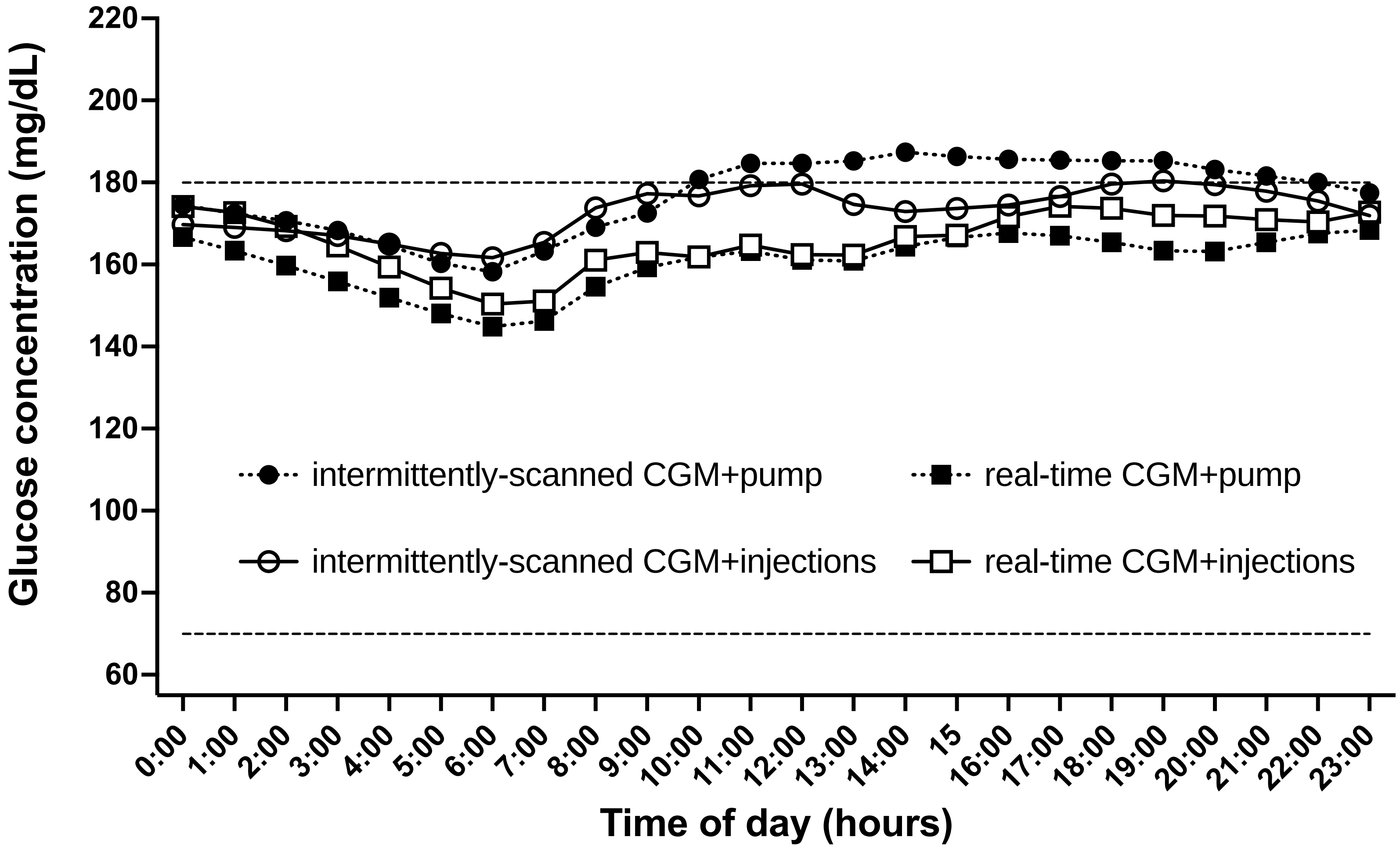

CGM – Continuous Glucose Monitoring

© 2023 Dovc K et al. *JAMA Network Open*.
