# Supplementary material for: Association of Achieving Time in Range Clinical Targets With Treatment Modality Among Youths With Type 1 Diabetes
Source: JAMA Netw Open. 2023 Feb 20;6(2):e230077. doi: 10.1001/jamanetworkopen.2023.0077 (PMC9941889; doi:10.1001/jamanetworkopen.2023.0077)
Supplement: Supplement 2. — Data Sharing Statement [file jamanetwopen-e230077-s002.pdf]

Data Sharing Statement

Dovc. Association of Achieving Time in Range Clinical Targets With Treatment Modality Among Youths With Type 1 Diabetes. *JAMA Netw Open*. Published February 20, 2023. doi:10.1001/jamanetworkopen.2023.0077

Data
Data available: Yes
Data types: Deidentified participant data
How to access data: klemen.dovc@mf.uni-lj.si
When available: With publication

Supporting Documents
Document types: None

Additional Information
Who can access the data: The datasets generated during and/or analyzed during the current study are available from the corresponding author on reasonable request.
Types of analyses: Meta-analysis of individual participant data, other reasonable request.
Mechanisms of data availability: After approval of a proposal
